# Supplementary material for: Acidification of Cytoplasm in Escherichia coli Provides a Strategy to Cope with Stress and Facilitates Development of Antibiotic Resistance
Source: Sci Rep. 2020 Jun 19;10:9954. doi: 10.1038/s41598-020-66890-1 (PMC7305162; doi:10.1038/s41598-020-66890-1)
Supplement: Supplementary file 1 — Supplementary information. [file 41598_2020_66890_MOESM1_ESM.pdf]

Acidification of Cytoplasm in *Escherichia coli* Provides a Strategy to Cope with Stress and Facilitates Development of Antibiotic Resistance

By

Esmeralda Z. Reyes-Fernández and Shimon Schuldiner*

Supplementary Material

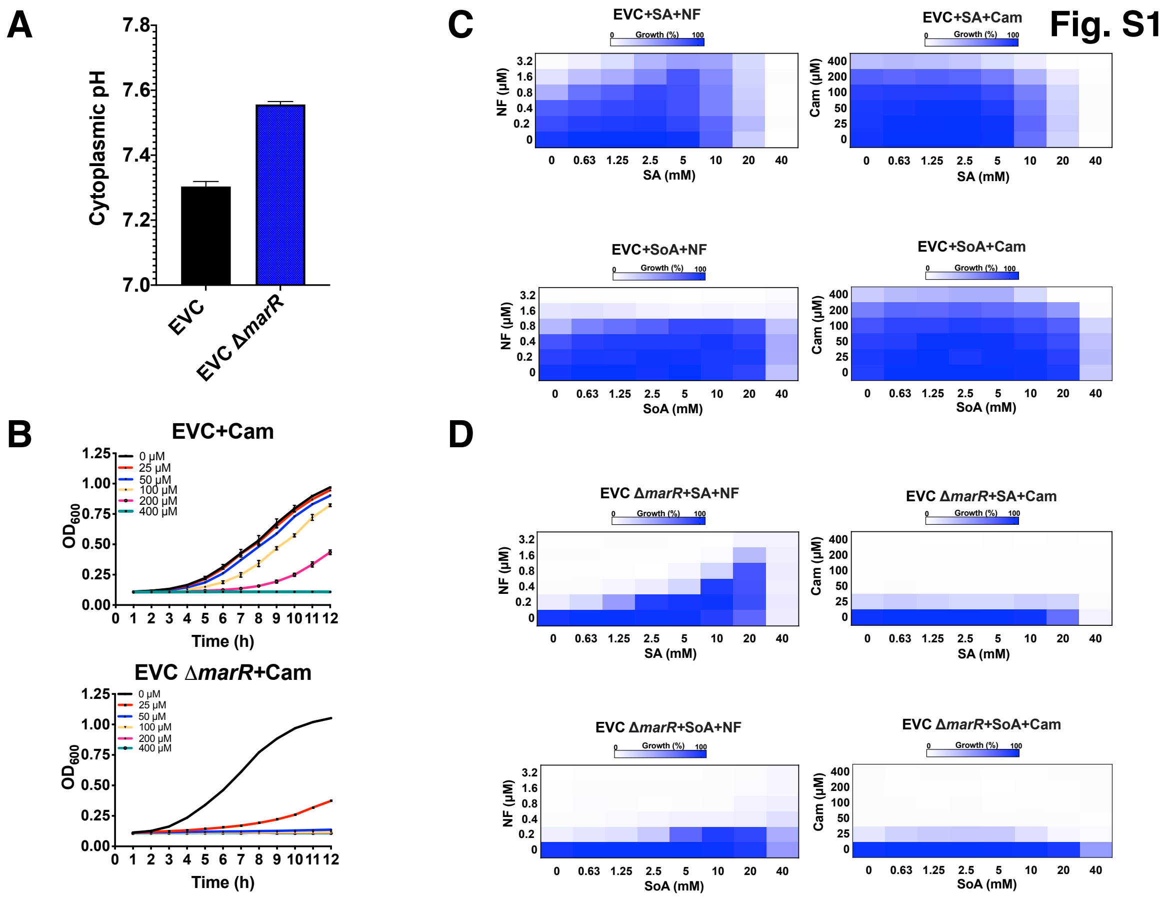

Figure S1. Inactivation of the *marRAB* operon in the highly resistant strain to Cam, EVC, causes an increase in the pH$_i$ and sensitivity of the strain to Cam and NF.

(A) Cytoplasmic pH of EVC, and its *marR* mutant. Cells were incubated for 1 h in MMA at 37°C, pH 7.4 **(B)** Growth curves of the EVC and EVC D*marR* incubated for 12 h in LB-KPi with increasing concentrations of Cam (25-400 mM). **(C)** Microdilution checkerboard analysis of the EVC or **(D)** EVC D*marR* incubated with weak acids in the presence of either NF or Cam. Growth was recorded after 12 hours of incubation of both strains.

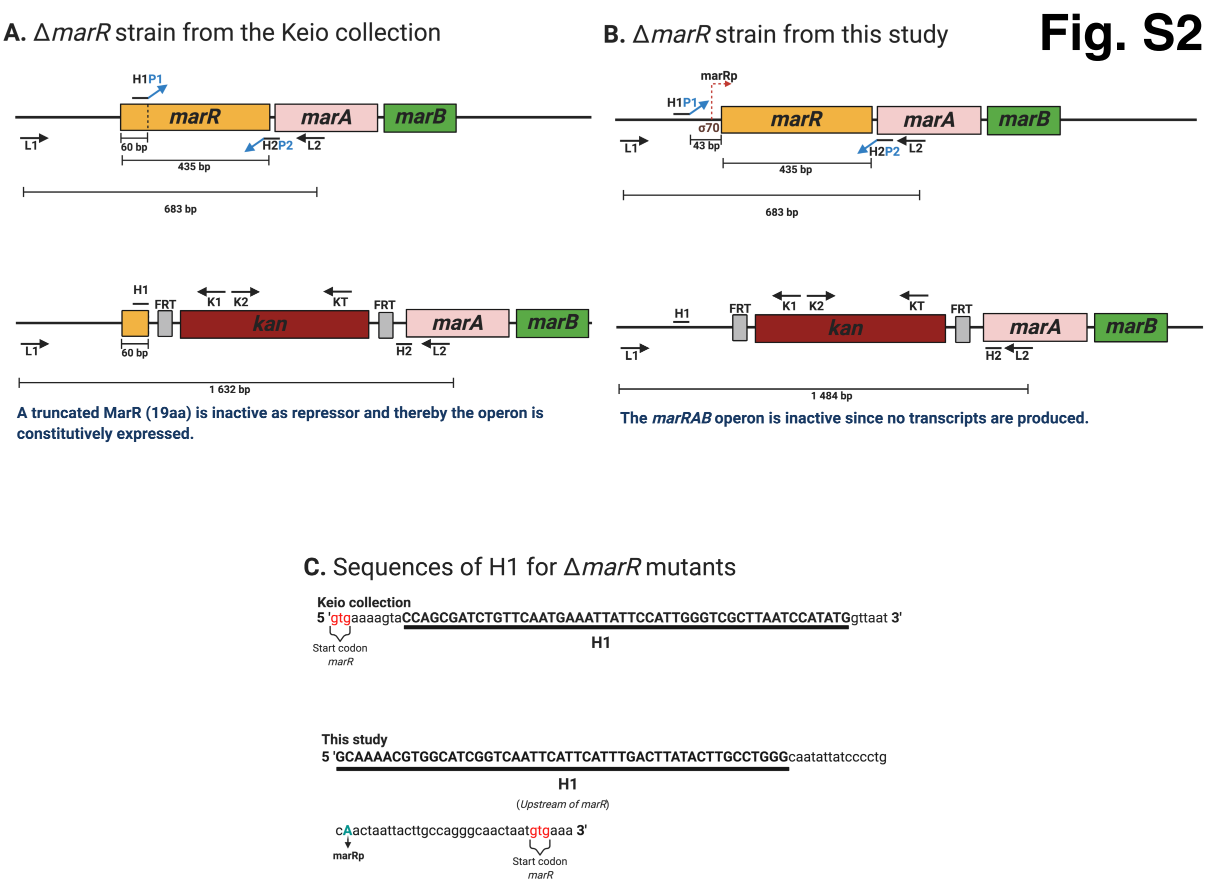

Figure S2. **Comparison of the Δ*marR* strain from the Keio collection (JW5248) and that generated in this study.** (A) Strategy to generate the Keio marR knockout strain. Inactivation of *marR* is achieved by the partial replacement of the marR ORF by the kanamycin resistance cassette (*kan*) as indicated in the figure. H1P1 primer was designed to leave intact the upstream region of *marR* and the first 60 bp of this gene (including the start codon). As a result, the 19 amino acids MarR repressor generated is inactive and the whole *marRAB* operon is constitutively expressed. (B) Deletion strategy of *marR* followed in this study. Inactivation of the entire *marRAB* operon was planned by deleting the whole *marR* ORF and 43 bp of its upstream sequence, which includes the *marRp* (promoter)/s70 factor binding site. Deletion of this region causes the inactivation of the operon since no transcripts are produced. The primer combination H1P1/H2P2 was used in both cases to amplify the kanamycin cassette (*kan*) from the pKD13 template. (C) Sequences of H1 component of the H1P1 primers used by the Keio collection and the present study. H1 sequences are highlighted in bold for both strategies. Start codon of *marR* is indicated in red. For full details of primer names, their sequences and the plasmids, see Tables S2 and S4 and the references therein.

Fig. S3

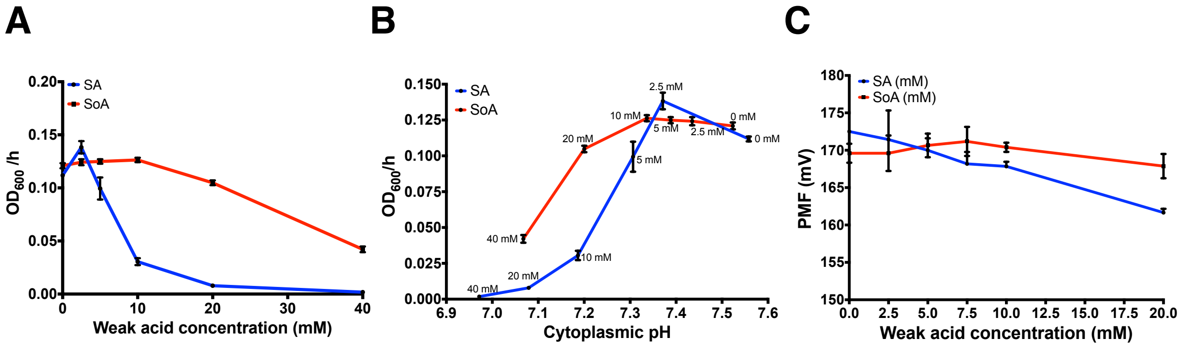

Figure S3. Effect of weak acids (SA and SoA) on the rate of growth (OD_{600}/h) and proton motive force (PMF) in the WT strain. (A) Correlation between the concentration of weak acid and the growth rate in the WT strain. Cells were grown in MMA + glycerol with the indicated concentrations of weak acids. **(B)** Cytoplasmic pH vs. growth rate of the WT strain in the presence of weak acids. Concentrations of SA (blue) and SoA (red) are indicated on each graph. **(C)** Proton motive force of the WT strain in the presence of SA or SoA. Proton motive force was estimated from equilibrium values of accumulation of [^{14}C] Lactose as described in Materials and Methods. Experiments were carried out in duplicates and repeated at least twice.

Fig. S4

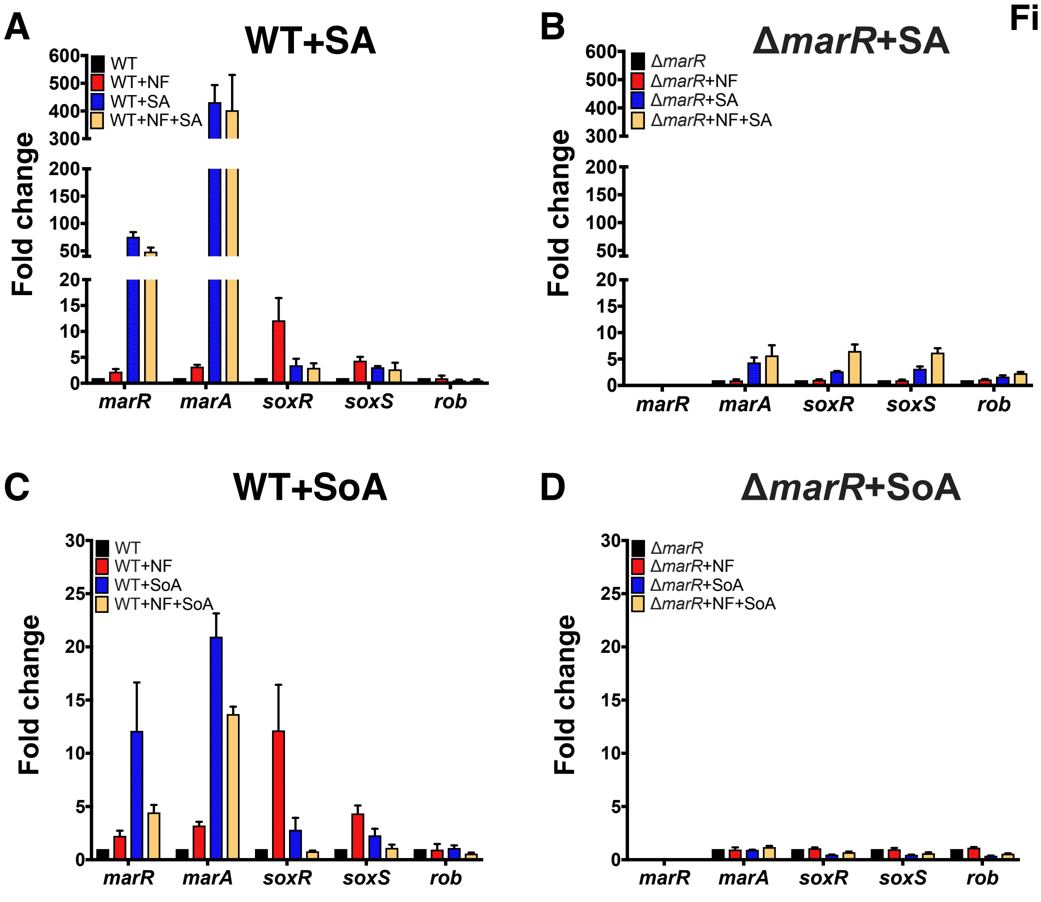

Figure S4. Real-time PCR analysis of the gene expression of *marR*, *marA*, *soxR*, *soxS* and *rob* in the WT and Δ*marR* strains exposed to NF, SA and SoA. (A) and (C) qRT-PCR analysis of *marR* and *marA, soxR*, *soxS* and *rob* mRNA levels in the WT strain after the transient exposure of the cells to SA **(A)**, SoA **(C)** or NF, as well as in combination of these compounds. **(B) and (D)** mRNA transcript levels of *marR*, *marA*, *soxR*, *soxS* and *rob* of the Δ*marR* under the same conditions as described above for the WT strain.

WT and mutant strains were grown in LB-K medium until OD_{600}=0.4 and exposed for 30 min to NF and weak acids. mRNA expression was normalized to the gene glyceraldehyde 3-phosphate dehydrogenase (*gapDH*).

Table S1. ***E. coli* strains used for this study.**

**Strain**	**Description**	**Reference**
WT	Wild-type strain (BW25113)	This study
Δ*marR*::*kan*	Deletion mutant of the *marR* gene in the background of the WT strain.	This study
Δ*soxR*::*kan*	Deletion mutant of the *soxR* gene in the background of the WT strain.	(1)
Δ*soxS*::*kan*	Deletion mutant of the *soxS* gene in the background of the WT strain.	(1)
Δ*rob*::*kan*	Deletion mutant of the *rob* gene in the background of the WT strain.	(1)
Δ*marR* Δ*soxR*::*kan*	Deletion mutant of the *soxR* gene in the background of the Δ*marR* strain.	This study
Δ*marR* Δ*soxS*::*kan*	Deletion mutant of the *soxS* gene in the background of the Δ*marR* strain.	This study
Δ*marR* Δ*rob*::*kan*	Deletion mutant of the *rob* gene in the background of the Δ*marR* strain.	This study
EV18*	NF resistant strain created by evolution *in vitro* (18 days habituation).	(2)
EV18Δ*marR*::*kan*	Deletion mutant of the *marR* gene in the background of the EV18 strain.	This study
EV18Δ*soxR*::*kan*	Deletion mutant of the *soxR* gene in the background of the EV18 strain.	This study
EV18Δ*soxS*::*kan*	Deletion mutant of the *soxS* gene in the background of the EV18 strain.	This study
EV18Δ*rob*::*kan*	Deletion mutant of the *rob* gene in the background of the EV18 strain.	This study
EV18Δ*marR* Δ*soxR*::*kan*	Deletion mutant of the *soxR* gene in the background of the EV18Δ*marR* strain.	This study
EV18Δ*marR* Δ*soxS*::*kan*	Deletion mutant of the *soxS* gene in the background of the EV18Δ*marR* strain.	This study
EV18Δ*marR* Δ*rob*::*kan*	Deletion mutant of the *rob* gene in the background of the EV18Δ*marR* strain.	This study

*Resequencing of this strain revealed a previously undetected mutation in *marR.* Starting at nucleotide 329 of marR, 38 bp deletion that results in permanent activation of the *mar* regulon.

Table S2. Plasmids used in this study.

Plasmid	Reference
pKD13	(3)
pKD46	(3)
pGFPR01	(4)
pKKmarA	This study

Table S3. Primers used for real-time PCR in this study.

Gene	Primer	Sequence (5'-3')	Tm (°C)
gapDH	*gapDH*_fw_qRTPCR	ACTTACGAGCAGATCAAAGC	52.6
	*gapDH*_rv_qRTPCR	AGTTTCACGAAGTTGTCGTT	52.4
soxR	*soxR*_fw_qRTPCR	ATCCGTAACAGCGGCAATCA	57.1
	*soxR*_rv_qRTPCR	TCGCACTTAACGTATGCCCT	56.5
soxS	*soxS*_fw_qRTPCR	GACCTGGGTTATGTCTCGCA	56.8
	*soxS*_rv_qRTPCR	TTACAGGCGGTGGCGATAAT	56.6
rob	*rob*_fw_qRTPCR	CGGCGAAAGCAGGTTATTCC	56.6
	*rob*_rv_qRTPCR	TTTCGACAAACGACGAGCAC	55.9
marA	*marA*_fw_qRTPCR	CATAGCATTTTGGACTGGAT	50.3
	*marA*_rv_qRTPCR	TACTTTCCTTCAGCTTTTGC	50.9
marR	*marR*_fw_qRTPCR	CTGTAAAGGCTGGGTGGAAAG	55.9
	*marR*_rv_qRTPCR	GGTCCTGGCCAACTAATTGATG	56

Table S4. **Primers used for generating the knock-out mutants used in this study.**

Primer*	Sequence (5'-3')	Tm (°C)
***marR*_H1P1**	GCAAAACGTGGCATCGGTCAATTCATTCATTTGACTTAT ACTTGCCTGGGATTCCGGGGATCCGTCGACC	71.4
***marR*_H2P2**	CGATCCAGTCCAAAATGCTATGAATGGTAATAGCGTCA GTATTGCGTCTGT GTAGGCTGGAGCTGCTTCG	70.4
***marR*_L1**	GCTAGCCTTGCATCGCATTGAA	58.4
***marR*_L2**	GACACTTTCTCCAGTGACAGTG	55.4
***soxR*_H1P1**	CTGTTGGGGAGTATAATTCCTCAAGTTAACTTGAGGTA AAGCGATTTATGATTCCGGGGATCCGTCGACC	69.2
***soxR*_H2P2**	AAAACAAACTAAAGCGCCCTTGTGGCGCTTTAGTTTTGT TCATCTTCCAGTGTAGGCTGGAGCTGCTTCG	71.3
***soxR*_L1**	GTCAATATGCTCGTCAATCC	51.1
***soxR*_L2**	CATACAATTAAAGCATCAAC	44.8
***soxS*_H1P1**	CCCCAACAGATGAATTAACGAACTGAACACTGAAAAGA GGCAGATTTATGATTCCGGGGATCCGTCGACC	70.1
***soxS*_H2P2**	GCGCGGGAGTTAACGCGCGGGCAATAAAATTACAGGC GGTGGCGATAATCTGTAGGCTGGAGCTGCTTCG	73.6
***soxS*_L1**	GGTTAGCAGCGCTTTAATGC	54.9
***soxS*_L2**	GCGTCGAAACTGAGGAGCAG	58.4
***rob*_H1P1**	AATTACCTGATGTCAGGTGCTCGTTGTTGAAAGGATGA GGATATTTTATGATTCCGGGGATCCGTCGACC	69.9
***rob*_H2P2**	GACGCCCCTGCATTAGATGAGCTGCAGCGTAAACGACG GATCGGAATCAGTGTAGGCTGGAGCTGCTTCG	73.1
***rob*_L1**	CGTCAAGCCCTAAAACATAC	51.2
***rob*_L2**	TAACTGTTCTATTTCGCGCG	53.1
**K1**	CAGTCATAGCCGAATAGCCT	54.1
**K2**	CGGTGCCCTGAATGAACTGC	59.2
**KT**	CGGCCACAGTCGATGAATCC	58.4

*The primers are named as described in (3).

1. T. Baba *et al.*, Construction of Escherichia coli K-12 in-frame, single-gene knockout mutants: the Keio collection. *Mol Syst Biol* **2**, 2006.0008 (2006).
2. Y. Shuster, S. Steiner-Mordoch, N. Alon Cudkowicz, S. Schuldiner, A Transporter Interactome Is Essential for the Acquisition of Antimicrobial Resistance to Antibiotics. *PLoS One* **11**, e0152917 (2016).
3. K. A. Datsenko, B. L. Wanner, One-step inactivation of chromosomal genes in Escherichia coli K-12 using PCR products. *Proc Natl Acad Sci U S A* **97**, 6640-6645 (2000).
4. K. A. Martinez, 2nd *et al.*, Cytoplasmic pH response to acid stress in individual cells of Escherichia coli and Bacillus subtilis observed by fluorescence ratio imaging microscopy. *Appl Environ Microbiol* **78**, 3706-3714 (2012).
